# Nodular fasciitis of the hand in a young athlete. A case report

**DOI:** 10.3109/03009734.2010.500746

**Published:** 2010-10-27

**Authors:** Hitomi Hara, Ikuo Fujita, Takuya Fujimoto, Keisuke Hanioka, Toshihiro Akisue, Masahiro Kurosaka

**Affiliations:** ^1^Department of Orthopaedic Surgery, Hyogo Cancer CenterJapan; ^2^Department of Pathology, Hyogo Cancer CenterJapan; ^3^Department of Orthopaedic Surgery, Kobe University Graduate School of MedicineJapan

**Keywords:** Hand, hand-ball player, nodular fasciitis

## Abstract

**Abstract:**

Nodular fasciitis is a rapidly growing mass, with high cellularity and mitotic activity, that can be both clinically and histologically misdiagnosed as a soft tissue sarcoma. Nodular fasciitis of the hand is an extremely rare condition. We report a 17-year-old male hand-ball player with nodular fasciitis in the dominant hand. The patient presented with a rapidly growing mass in his right hand and no history of major trauma. On physical examination, a painful mass measuring 2 cm in diameter was observed in the first web space. Magnetic resonance imaging (MRI) demonstrated a subcutaneous mass with isointensity on T1-weighted images and inhomogeneous high intensity on T2-weighted images. The lesion was inhomogeneously enhanced after intravenous administration of gadolinium. Moreover, thallium-201 scintigraphy showed high uptake at the early phase and no wash-out at the delayed phase. We performed an excisional biopsy. The mass was present subcutaneously and adhered to the interosseous muscle fascia. Although a pathological examination by frozen section during surgery showed a low-grade spindle cell sarcoma, the final histological diagnosis was nodular fasciitis. There was no evidence of local recurrence at the recent follow-up 2 years after the operation. We speculate that repeated small injuries as a result of sports activities played an important causative role in the nodular fasciitis.

## Introduction

Nodular fasciitis is a rapidly growing benign, self-limiting, reactive lesion, which due to clinical findings and pathologic appearance can be mistaken for a soft tissue sarcoma. This lesion is commonly found in the forearm and is extremely rare in the hand. The precise cause of nodular fasciitis is unknown, but despite a benign clinical behavior injury or infection is possible.

We present a case of nodular fasciitis in the hand of a hand-ball player. In our patient, thallium-201 scintigraphy showed high uptake at the early phase and no wash-out at the delayed phase in a so-called malignant pattern. These scintigraphy findings have not been previously reported in nodular fasciitis. To our knowledge, this is the first report of nodular fasciitis occurring in the hand of an athlete.

## Case report

**Figure 1.**
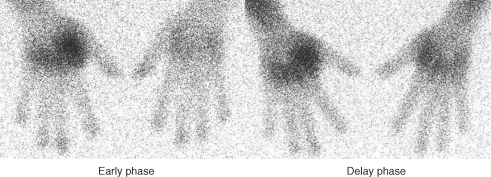
Radiographs of the right hand show an increase in soft tissue shadow in the first web space.

**Figure 2.**
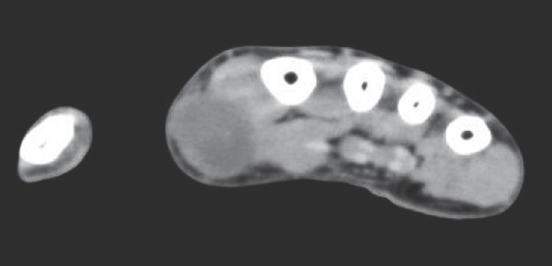
On computed tomography, the 20 mm in diameter mass shows a lower density than the muscle, and its margins are comparatively clear.

**Figure 3.**
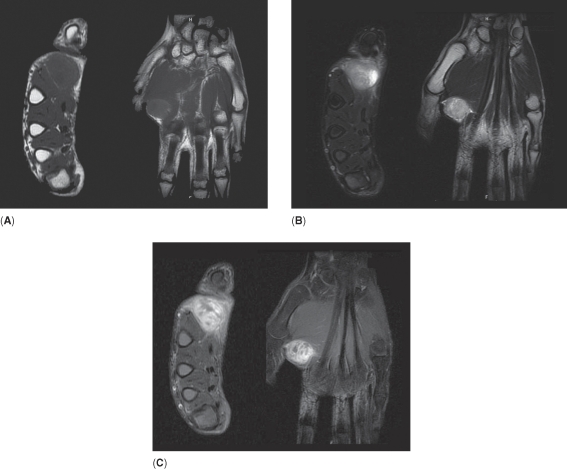
On magnetic resonance imaging (MRI), the mass has low intensity on T1-weighted images (A) and is heterogeneously hyperintense on T2-weighted images (B). After intravenous gadolinium, the mass is enhanced heterogeneously on T1-weighted images (C).

**Figure 4.**
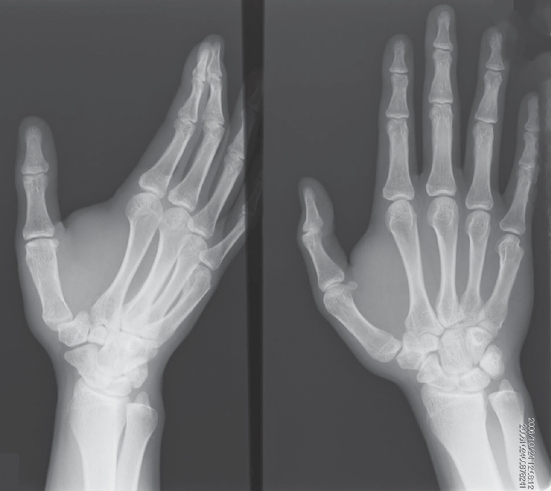
Thallium-201 scintigraphy shows high uptake in the early phase and no wash-out on the delayed phase.

A 17-year-old male high school hand-ball player noticed an approximately 1-cm mass in the first web space of his dominant right hand 1 month before presentation at our hospital. He sought medical attention because the mass became rapidly and painfully enlarged, doubling during the 4-week interval from first observation. He belonged to a high school hand-ball club and played every day. He had not suffered any infection or major trauma, and there was no relevant family history. On physical examination at our hospital, a painful mass measuring 2 cm in diameter was observed in the first web space. The mass was mobile, elastic-hard, and tender to palpation with a smooth surface and clear margin. No local heat and redness on the mass were present. Neurovascular examinations including Tinel's sign were normal. His hematological profile was unremarkable. Radiographs of the right hand showed an increased soft tissue shadow in the first web space (Figure 1). On computed tomography, the mass, 20 mm in diameter, showed a lower density than muscle (CT value was 30 to 50 H.U.), with a comparatively clear margin (Figure 2). There was no invasion or calcification. Magnetic resonance imaging (MRI) confirmed a smooth surface mass measuring 20 × 20 × 16 mm in the first web space of the patient's right hand. The mass had low intensity on T1-weighted images (Figure 3A) and was heterogeneously hyperintense on T2-weighted images (Figure 3B). After intravenous injection of the gadolinium—diethylenetriamine penta-acetic acid—the mass was enhanced heterogeneously on T1-weighted images (Figure 3C). Thallium-201 scintigraphy showed high uptake at the early phase and no wash-out at the delayed phase (Figure 4).

**Figure 5.**
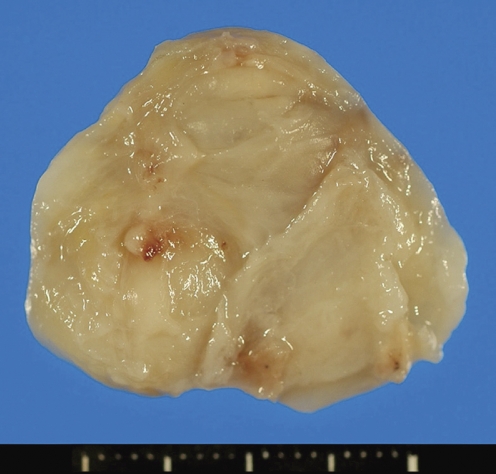
On macroscopy, the solid mass measures 25 mm in diameter.

**Figure 6.**
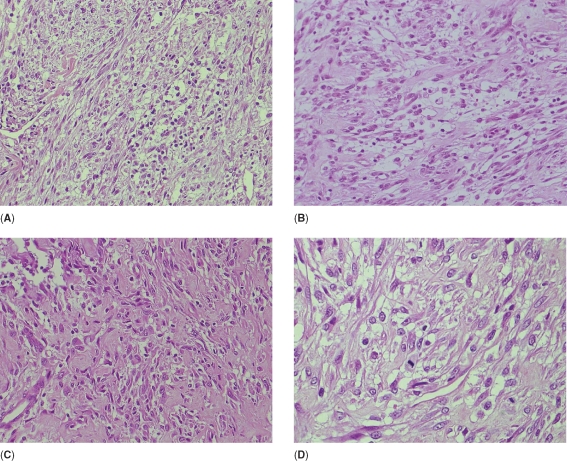
A: The specimen shows vascular hyperplasia and infiltration of inflammatory cells (hematoxylin and eosin, ×100). B: The spindle cells formed S- or C- shaped fascicles in the highly cellular area (hematoxylin and eosin, ×200). C: In the poor cellular area, plump and spindle cells are present between the hyalinization of fibrous stroma (hematoxylin and eosin, ×200). D: little variation in size and shape of the nuclei. Mitotic activity ranges from 2 to 3 mitotic figures per 10 high-power fields, but atypical mitoses are hardly seen (hematoxylin and eosin, ×400).

Eleven days after the first visit, the mass was even more enlarged measuring 25 mm in diameter. We performed an excision biopsy (Figure 5). A well circumscribed white homogeneous mass was present subcutaneously and adhered to the interosseous muscle fascia. Intraoperative examination by frozen section showed a low-grade spindle-cell sarcoma. Histological examination by permanent section showed vascular hyperplasia and infiltration of inflammatory cells (Figure 6A). The spindle cells formed S- or C-shaped fascicles in the highly cellular area (Figure 6B). In the poor cellular area, plump and spindle cells were present between the hyalinization of fibrous stroma (Figure 6C). There was little variation in the size and shape of the nuclei. Mitotic activity ranged from two to three mitotic figures per ten high-power fields, but atypical mitoses could hardly be detected (Figure 6D). Immunohistochemical studies showed alpha-SMA and HHF-35, but no desmin; CA5.2, S-100, CD34, and c-kit were positive. MIB-1 labeling index measured 5%–10%. P53-positive cells were about 50%. From these findings, the final diagnosis of nodular fasciitis was made. The patient was able to restart hand-ball 2 months after the operation. There has been no local recurrence at the recent follow-up 2 years after operation.

## Discussion

Nodular fasciitis is a benign reactive lesion first reported by Konwaler et al. in 1955 as a subcutaneous pseudosarcomatous fibromatosis (1). It is a rapidly growing mass occurring in all age groups but most often in young adults between 20 and 40 years of age but with no racial or gender predilection. In about 10%–50% of patients there is associated pain or tenderness (2,3). It is usually subcutaneous, although occasional cases involve muscle and fascia. Although nodular fasciitis can occur virtually anywhere in the body, it is commonly found in the volar aspect of the forearm, next in frequency is the chest wall and back, followed by the neck and head (2,4). This disorder is less common in the hand. A large series of nodular fasciitis showed only 0%–2% of occurrences in the hand (2,4,5). Since Brimhall et al. first made a detailed case report of nodular fasciitis of the hand in 1989 (6), only 13 cases (including the current case) have been reported in the English literature as summarized in Table I. The age and size distribution of nodular fasciitis of the hand is similar to those at general sites. There has been no mention of occupation nor sports activity in all published cases.

**Table I.** Summary of previously reported cases of nodular fasciitis of hand.

Authors (ref)	Year	Site	Age (yrs)	Preoperative diagnosis	Treatment	Size	Previous history
Brimhall et al. (6)	1989	palm	5.5	neoplasm with spindle-shaped cells and mucin deposition	excision	1.5 cm	trauma
Rankin et al. (3)	1991	palm	39	N/A	excision	1 cm	no
Katz et al. (7)	2001	first web space	38	N/A	excision	2 cm	no
Donner et al. (18)	2002	palm	48	N/A	excision	2 cm	trauma
Bau et al. (19)	2003	palm	53	N/A	excision	N/A	N/A
Singh et al. (20)	2004	thumb	43	fibromatosis, fibrous histiocytoma, nodular fasciitis, giant cell tumor of flexor sheath	excision	2 cm	no
Kijima et al. (5)	2005	index finger	30	N/A	excision	2 cm	N/A
Plaza et al. (15)	2005	palm	51	clinical diagnosis: soft tissue sarcoma; diagnosis by FNAB: spindle-cell sarcoma	wide resection	1.7 cm	trauma
		index finger	40	clinical diagnosis: epithelioid sarcoma; diagnosis by FNAB: spindle-cell sarcoma	wide resection	3.8 cm	no
		palm	42	clinical diagnosis: sarcoma; diagnosis by FNAB: nodular fasciitis	excision	2.5 cm	fracture
Ikeda et al. (21)	2005	palm	42	N/A	excision with ulnar nerve	2 cm	no
Sailon et al. (22)	2008	little finger	52	N/A	excision	2.3 cm	no
Hara et al.	2010	first web space	17	nodular fasciitis	excision	2 cm	hand-ball player

FNAB = fine-needle aspiration biopsy; N/A = data are not available.

The macroscopic appearance of nodular fasciitis is solitary round to oval nodules, well circumscribed, and usually measuring less than 2 cm in diameter; only 8% are larger than 4 cm (2). It is often initially misdiagnosed as sarcoma because of its rapidly growing nature of 1 month or less in duration. The etiology of nodular fasciitis remains unclear, but it is considered to be a self-limiting reactive lesion and not a true neoplasm as some cases have occurred after trauma or infection. Bernstein and Lattes described a recognized history of trauma in 5 of 134 cases (2). Although the number of patients with trauma was low, they described the possibility of minor trauma causing this reactive disorder. In the reported hand cases, 5 of 13 cases (38.5%), including our patient, had a history of trauma (Table I). Nodular fasciitis of the hand seems to have a close association with trauma compared to other locations. In the current case, the patient played hand-ball every day. Although he had no history of major injury, catching or throwing the ball could have induced repeated minor trauma on the affected hand and may be connected to the cause of the nodular fasciitis.

The features of nodular fasciitis on MR imaging are non-specific. The lesions appear as well circumscribed, round to oval masses (7). Literature descriptions of the signal intensity of the condition, including the contrast enhancement pattern, vary (7–11). On MRI, intramuscular lesions appear mildly inhomogeneous and hyperintense to skeletal muscle on T1-weighted spin-echo images; whereas on T2-weighted spin-echo images, the lesions are relatively homogeneous with hyperintense signal to subcutaneous fat. Subcutaneous lesions, typically more fibrous than intramuscular lesions, are markedly hypointense to skeletal muscle on all spin-echo sequences and appear homogeneous in texture (7,10–12). High cellularity and micro-vessel density may directly influence the early enhancement after intravenous gadolinium injection and compact cellularity with a prominent capillary network. A myxoid pattern may be responsible for the enhancement on MRI (12). In our patient, thallium-201 scintigraphy showed high uptake at the early phase and no wash-out at the delayed phase, so-called malignant pattern, but these scintigraphy findings have not been previously reported in nodular fasciitis. Kessels et al. reported that nodular fasciitis was detected by positron emission tomography with 18F-fluorodeoxyglucose (18-FDG-PET) (13). However when thallium-201 scintigraphy or 18-FDG-PET shows a high uptake similar to sarcoma, nodular fasciitis should be included in the differential diagnosis.

Histologically, the lesion is composed of plump but regular spindle-shaped fibroblasts or myofibroblasts lacking nuclear hyperchromasia and pleomorphism. The lesion may be highly cellular. Although mitotic figures are fairly common, atypical mitoses are almost never seen. Five important histologic features of nodular fasciitis aid in diagnosis, including spindle-shaped fibroblasts, clefts separating the fibroblasts, extravasated erythrocytes, interstitial mucoid material, and a loosely textured ‘feathery’ pattern of the mucopolysaccharide ground substance (3). Immunostains for vimentin and alpha-SMA are usually positive, but desmin, cytokeratin and S-100 are typically negative. Because of common misdiagnosis, incisional or excisional biopsy is usually required for a definitive diagnosis. Montgomery and Meis verified that correct tissue diagnosis of nodular fasciitis was made in fewer than 50% of cases submitted to the Armed Forces Institute of Pathology (14). Plaza et al. reported that nodular fasciitis remained a difficult diagnosis by fine-needle aspiration biopsy (FNAB), particularly when it occurred in locations such as the hand (15). Careful histological examination is important to avoid radical surgery. In this case, the operative frozen section was insufficient for a diagnosis of nodular fasciitis. We recommend excisional or incisional biopsy, and a permanent histological section, including immunohistochemical study to confirm the diagnosis. Most lesions are effectively treated by local excision, as reflected by a recurrence rate of 1% to 2% (2,4,16,17). Recurrence soon after excision has been associated with incomplete excision of the lesion (7).

The current report documents a rare case of nodular fasciitis in the hand. Particularly, nodular fasciitis in the hand of a sports player has never been reported. Repeated minor injury to the hand during catching or throwing the ball may have caused the nodular fasciitis. Nodular fasciitis should be added to the differential diagnosis of ball game players who present with a soft-tissue mass in the hand.
